# Supplementary material for: Impact of Biogenic Structures of the Soil-Nesting Ants Lasius niger and Lasius flavus on the Soil Microarthropod Community in Urban Green Spaces
Source: Insects. 2025 Oct 17;16(10):1058. doi: 10.3390/insects16101058 (PMC12564664; doi:10.3390/insects16101058)
Supplement: Supplementary file 1 [file insects-16-01058-s001.zip › insects-3853554-supplementary.pdf]

Article

Impact of Biogenic Structures of the Soil-Nesting Ants *Lasius niger* and *Lasius flavus* on the Soil Microarthropod Community in Urban Green Spaces

Maria Sterzyńska [1,*], Dariusz J. Gwiazdowicz [2], Paweł Nicia [3], Paweł Zadrożny [3], Gema Trigos-Peral [1] and Mohamed W. Negm [4]

[1] Museum and Institute of Zoology PAS, Twarda 51/55, 00-818 Warsaw, Poland; MS e-mail: majka@miiz.waw.pl; GT-P e-mail: gtrigs@miiz.waw.pl

[2] Department of Forest Entomology and Pathology, Poznań University of Life Sciences, Wojska Polskiego, 71c, 60-625 Poznań , Poland, e-mail: dariusz.gwiazdowicz@up.poznan.pl

[3] Department of Soil Science and Agrophysics, University of Agriculture in Kraków, Al. Mickiewicza 21, 31-120 Kraków, Poland, e-mail: rrnicia@cyf-kr.edu.pl, pawel.zadrozny@urk.edu.pl

[4] Department of Plant Protection, Faculty of Agriculture, Assiut University, Assiut 71526, Egypt, e-mail: waleednegm@yahoo.com

* Correspondence: majka@miiz.waw.pl

Supplementary Materials: The following supporting information can be downloaded at: https://www.mdpi.com/article/doi/s1. Figure S1: Distribution pattern of Collembola species within ant mounds of LN and LF. CCA model calculated with log(x+1) transformed data. pCCA analysis with site as covariate; model calculated with log(x+1) transformed data. Species names and acronyms are given in Supplementary Table S4; Figure S2: Distribution pattern of Mesostigmata species within ant mounds of LN and LF. CCA model calculated with log(x+1) transformed data. pCCA analysis with site as covariate; model calculated with log(x+1) transformed data. Species names and acronyms are given in Supplementary Table S5; Table S1: Loadings of soil characteristics from PCA from *L. niger* + *L. flavus* mounds and control; Table S2: Loadings of soil characteristics from PCA from *L. niger* and *L. flavus* mounds; Table S3:Effect of *L. flavus* and *L. niger* ant mounds (AM), season(S) and site (Sl) indicated by RDA and pRDA in soil microarthropods taxonomic composition (i.e. using high taxonomic ranks of soil microarthropods); Table S4: Collembola species composition, abundance and diversity parameters in the *L. flavus* (LF) and *L. niger* (LN) mounds; Table S5: Mesostigmata species composition, abundance and diversity parameters in the *L. flavus* (LF) and *L. niger* (LN) ant mounds.

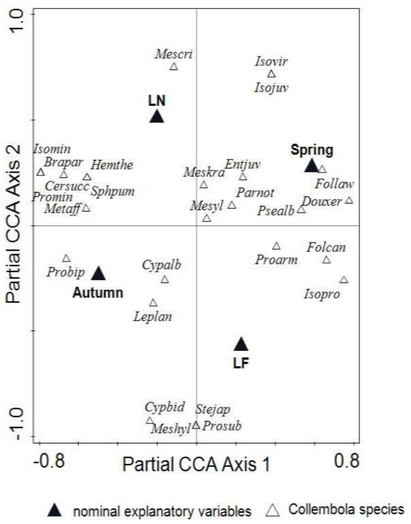

Figure S1. Distribution pattern of Collembola species within ant mounds of LN and LF. CCA model calculated with log(x+1) transformed data. pCCA analysis with site as covariate; model calculated with log(x+1) transformed data. Species names and acronyms are given in Supplementary Table S4.

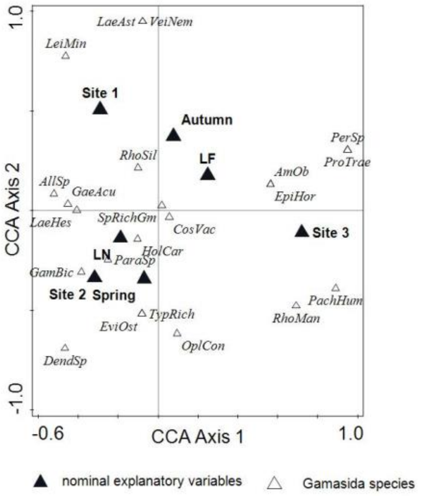

Figure S2. Distribution pattern of Mesostigmata species within ant mounds of LN and LF. CCA model calculated with log(x+1) transformed data. pCCA analysis with site as covariate; model calculated with log(x+1) transformed data. Species names and acronyms are given in Supplementary Table S5.

Table S1. Loadings of soil characteristics from PCA from *L. niger* + *L. flavus* mounds and control

PCA component	Axis 1	Axis 2
pH H20	-0.35	0.07
TK (g kg-1)	0.60	0.79
TP (g kg-1)	0.92	0.01
TN (g kg-1)	0.95	-0.24
TC (g kg-1)	0.95	-0.25
C/N ratio	0.40	-0.20
Moisture (%)	-0.04	-0.33
Tsoil (ºC)	-0.58	0.22
EC (µS cm-1)	0.13	0.04

Table S2. Loadings of soil characteristics from PCA from *L. niger* and *L. flavus* mounds.

PCA component	Axis 1	Axis 2
pH H20	-0.28	0.21
TK (g kg-1)	0.60	-0.79
TP (g kg-1)	0.88	0.17
TN (g kg-1)	0.97	0.08
TC (g kg-1)	0.97	0.09
C/N ratio	0.61	0.11
Moisture (%)	-0,30	-0.33
Tsoil (ºC)	-0.62	-0.18
EC (µS cm-1)	0.36	0.53

Table S3. Effect of *L. flavus* and *L. niger* ant mounds (AM), season(S) and site (Sl) indicated by RDA and pRDA in soil microarthropods taxonomic composition (i.e. using high taxonomic ranks of soil microarthropods)

Explanatory variables	Covariates	Explained variance (%)	Explained variance (adj. %)	pseudo-F	p-value
AM + S + Sl		14.1	3.0	1.3	0.258
AM	T+S	6.9	3.9	2.3	0.074
T	AM+ S	6.5	3.5	2.2	0.074
S	T+AM	2.0	0.0	0.3	0.950

Table S4. Collembola species composition and diversity parameters in the *L. flavus* (LF) and *L. niger* (LN) mounds.

No	Species	Abbreviation	LN mound	LF mound
**1**	*Brachystomella parvula* (Schaeffer, 1896)	Brapar	*	
**2**	*Ceratophysella succinea* (Gisin, 1949)	Cersucc	*	
**3**	*Cyphoderus albinus* Nicolet, 1842	Cypalb	*	*
**4**	*Cyphoderus bidenticulatus* Parona, 1888	Cypbid		*
**5**	*Doutnacia xerophila* Rusek, 1974	Douxer	*	*
**6**	*Folsomia candida* Willem, 1902	Folcan		*
**7**	*Folsomia lawrencei* Rusek, 1984	Follaw	*	*
**8**	*Hemisotoma thermophila* (Axelson, 1900)	Hemthe	*	
**9**	*Isotoma viridis* Bourlet, 1839	Isovir	*	
**10**	*Isotomiella minor* (Schaeffer, 1896)	Isomin	*	
**11**	*Isotomodes productus* (Axelson, 1906)	Isopro		*
**12**	*Lepidocyrtus lanuginosus* (Gmelin, 1788)	Leplan	*	*
**13**	*Mesaphorura critica* Ellis, 1976	Mescri	*	
**14**	*Mesaphorura hylophila* Rusek, 1982	Meshyl		*
**15**	*Mesaphorura krausbaueri* Börner, 1901	Meskra	*	*
**16**	*Mesaphorura sylvatica* (Rusek, 1971)	Mesyl	*	*
**17**	*Metaphorura affinis* (Börner, 1903)	Metaff	*	*
**18**	*Parisotoma notabilis* (Schaeffer, 1896)	Parnot	*	*
**19**	*Proisotoma minima* (Absolon, 1901)	Promin	*	
**20**	*Proisotomodes bipunctatus* (Axelson, 1903)	Probip	*	*
**21**	*Protaphorura armata* (Tullberg, 1869)	Proarm	*	*
**22**	*Protaphorura subarmata* (Gisin, 1957)	Prosub		*

**23**	*Pseudosinella alba* (Packard, 1873)	Psealb	*		*	
**24**	*Sphaeridia pumilis* (Krausbauer, 1898)	Sphpum	*			
**25**	*Stenaphorura japygiformis* Absolon, 1900	Stejap			*	
	Entomobryidae juv.	Entjuv	*		*	
	Isotomidae juv.	Isojuv	*			
			mean	SD	mean	SD
	Species richness (S)		2.78	1.73	2.50	2.18
	Shannon's diversity index (H′)		0.731	0.504	0.615	0.539

Table S5. Mesostigmata species composition, abundance and diversity parameters in the *L. flavus* (LF) and *L. niger* (LN) ant mounds.

No	Species	Abbreviation	LN mound		LF mound	
1	*Alloparasitus* sp.	All.sp	*			
2	*Amblyseius obtusus* (C.L. Koch, 1839)	Am.ob	*			
3	*Cosmolaelaps vacua* (Michael, 1891)	Cos.vac	*		*	
4	*Dendrolaelaps* sp.	Dend.sp	*			
5	*Epicriopsis horidus* (Kramer, 1876)	Epi.hor	*			
6	*Eviphis ostrinus* (C.L. Koch, 1836)	Evi.ost			*	
7	*Gaeolaelaps aculeifer* (Canestrini, 1883)	Gae.acu	*			
8	*Gamasellodes bicolor* (Berlese, 1918)	Gam.bic	*		*	
9	*Holoparasitus calcaratus* (C.L. Koch, 1839)	Hol.car	*			
10	*Laelaspis astronomica* (C.L. Koch, 1839)	Lae.ast			*	
11	*Laelaspis heselhausi* (Oudemans, 1912)	Lae.hes	*			
12	*Leioseius minusculus* (Berlese, 1905)	Lei.min	*			
13	*Oplitis conspicua* (Berlese, 1903)	Opl.con	*			
14	*Pachyseius humeralis* Berlese, 1910	Pach.hum			*	
15	*Paragamasus* sp.	Para.sp	*		*	
16	*Pergamasus* sp.	Per.sp			*	
17	*Prozercon traegardhi* (Halbert, 1923)	Pro.trae			*	
18	*Rhodacarellus silesiacus* Willmann, 1935	Rho.sil	*		*	
19	*Rhodacarus mandibularis* Berlese, 1921	Rho.man	*		*	
20	*Typhlodromus richteri* Karg, 1970	Typ.rich			*	
21	*Veigaia nemorensis* (C.L. Koch, 1839)	Vei.nem			*	
			mean	SD	mean	SD
	Species richness (S)		1,61	1.38	1.33	0.97
	Shannon's diversity index (H′)		0.414	0.488	0.327	0.390
